# Metabolic changes in pomegranate fruit skin following cold storage promote chilling injury of the peel

**DOI:** 10.1038/s41598-021-88457-4

**Published:** 2021-04-28

**Authors:** Ravi Singh Baghel, Alexandra Keren-Keiserman, Idit Ginzberg

**Affiliations:** 1 Institute of Plant Sciences, Agricultural Research Organization, Volcani Center, 68 HaMacabim Road, P.O. Box 15159, 7505101 Rishon LeZion, Israel; 2 Present Address: Biological Oceanography Division, CSIR-National Institute of Oceanography, Dona Paula, Goa 403004 India

**Keywords:** Molecular biology, Physiology, Plant sciences

## Abstract

Pomegranate cv. ‘Wonderful’ fruit are susceptible to chilling injuries of the peel (CIp) when stored at 7 °C in modified-atmosphere bags for more than 3 months. The damage, manifested as superficial browning, is restricted to the fruit skin, i.e., the outer colored layer of the peel. To characterize possible causes of CIp development, fruit were collected at early harvest—when the premature fruit are poorly colored and susceptible to CIp development, and at late harvest—when mature fruit have fully red skin and less susceptibility to CIp. Skin samples were collected on day of harvest and at different time points during storage. Anatomical study of skin with CIp disorder showed a broken cuticle layer with underlying degenerated cells. A high total phenol content, which is associated with high antioxidant capacity, was not sufficient to prevent the development of CIp in the premature fruit. The concentration of punicalagin was the same for premature and mature skin at harvest and during storage, and therefore not associated with CIp development in the premature fruit skin. Furthermore, the expression of antioxidant-related genes *CAT2*, *SOD* and *GR2* was similar for both premature and mature fruit skin. Poor pigmentation of the premature fruit skin and chilling-induced downregulation of key anthocyanin-biosynthesis genes were associated with CIp development. High total phenol concentration combined with high expression of the gene encoding PPO was also associated with CIp; however, high expression ratio of *PAL* to *PPO* was found in mature skin, and may be associated with reduced CIp disorder. The results presented suggest future possibilities for controlling the CIp phenomenon.

## Introduction

Pomegranate (*Punica granatum* L.) fruit is well known for its health-beneficial metabolites^1–4^, and there is extensive demand for it in global markets. The main cultivar in Israel is ‘Wonderful’. Harvesting lasts 3 weeks, and then the fruit is sorted, packed and stored. High premium pomegranate is intended for export, which necessitates prolonged storage of the fruit. For that purpose, the fruit is packed in modified-atmosphere (MA) bags and stored at 7 °C until shipment. Up to 3 months of storage, the fruit maintains its visual, organoleptic and nutritive quality. However, when storage period is extended up to 5 months, fruit appearance deteriorates even if maintained in MA bags at 7 °C. The response of the pomegranate peel to reduced temperatures is usually referred to as ‘husk scald’ (HS), however, later on in this work we show that this term refers to two different symptoms that develop on the surface of the fruit during the cold storage.

In the literature, HS has been studied in pomegranate accession lines and under different storage conditions^5–8^. HS is superficial browning that is restricted to the peel, and has been suggested to result from the oxidation of *o*-dihydroxyphenols^9^. However, its causes are not yet clear. The HS phenomenon differs from chilling damage to the inner fruit tissues that is mostly manifested as internal browning of the membranous walls and the white spongy tissue surrounding the arils^10,11^. Chilling injury (CI) of inner tissues is induced after a short period of storage (2 weeks) at 1 °C, and browning becomes apparent only after transfer of the fruit to shelf-life conditions at 20 °C.

The pomegranate fruit peel contains a high concentration of two groups of polyphenols, anthocyanins (AT)—the main pigments in pomegranate, and hydrolyzable tannins (HT), both are synthesized from an intermediate of the shikimate pathway^12,13^. The AT and HT confer strong antioxidant activity and are considered to be potential protectants from chilling damage^7,14,15^.

The biochemical pathway and genetic control of AT production have been well characterized in numerous plant species and are considered to be highly conserved among different species in the plant kingdom^16^. Common flavonoid precursors are catalyzed by chalcone synthase (CHS), chalcone isomerase (CHI), flavanone 3-hydroxylase, dihydroflavonol 4-reductase (DFR), and leucoanthocyanidin dioxygenase. The expression of DFR is correlated with pomegranate color and the timing of color appearance during fruit development^17^.

Six AT pigments have been identified in pomegranate fruit: mono- and diglucosides of cyanidin (red pigments), delphinidin (purple pigments) and pelargonidin (orange pigments)^18–21^. The transcription factor *PgWD40* has been shown to regulate the expression of downstream structural genes involved in AT biosynthesis in pomegranate fruit. Its expression level was correlated with the expression level of the structural enzyme DFR and with total cyanidin level in the skin^17^.

Only traces of the HT-specific pathway are known. It branches from the shikimate pathway, with shikimate dehydrogenase as the first committed step catalyzing gallic acid^12^. This is followed by production of β-glucogallin and then pentagalloyl glucose^22^. Further oxidative modifications of pentagalloyl glucose lead to the formation of gallotannins and ellagitannins (e.g., punicalagin in pomegranate)^22–24^.

The correlation between antioxidant capacity, AT and HT levels, and the resistance of the pomegranate peel to HS development is not yet conclusive^7,8^. This could be due to variations in the response among different accession lines, or to metabolite analyses performed with the whole peel. In the present study, we used cv. ‘Wonderful’ which bears large, late-season fruit that ripen in October and is “sweet–sour” in taste^2^. Only the colored outer layer of the peel, the skin, was analyzed, as the development of chilling-induced HS is limited to that layer. The effect of skin color/maturation on tolerance to chilling conditions was tested in early- and late-harvested fruit, differing in pigment intensity. The fruit were stored in Xtend bags, the current commercial practice for extended storage time^25^. The major AT and HT metabolites in the pomegranate skin were profiled during storage, along with a determination of total polyphenol content (TPC), skin antioxidant capacity, and related gene expression.

## Materials and methods

### Plant material and experimental design

Pomegranate cv. ‘Wonderful’ fruit were collected from a commercial orchard at Kibbutz Tsor’a, located in the central region of Israel (31° 45′ 37″ N 34° 57′ 48″ E). The use of pomegranate was carried out in accordance with relevant guidelines and regulation. Within 24 h of harvest, fruit were washed with the fungicide Scholar (Syngenta, USA) and stored at 7 °C in Xtend MA bags for pomegranates (http://www.stepac.com/products.asp?siteid=971&pageid=1497; StePac L.A. Ltd., Israel)^25^.

Two experiments were conducted. In experiment 1, fruit harvested from commercial plots was sorted at the packing house for export quality, and was stored for 4 months at 7 °C in Xtend bags (bin bags for ~ 400 kg, code: RIM2.5), until CI of the peel were apparent. At that stage, fruit peel was sampled for the analyses detailed below, at three biological replicates, four fruit per replicate.

**Figure 1:**
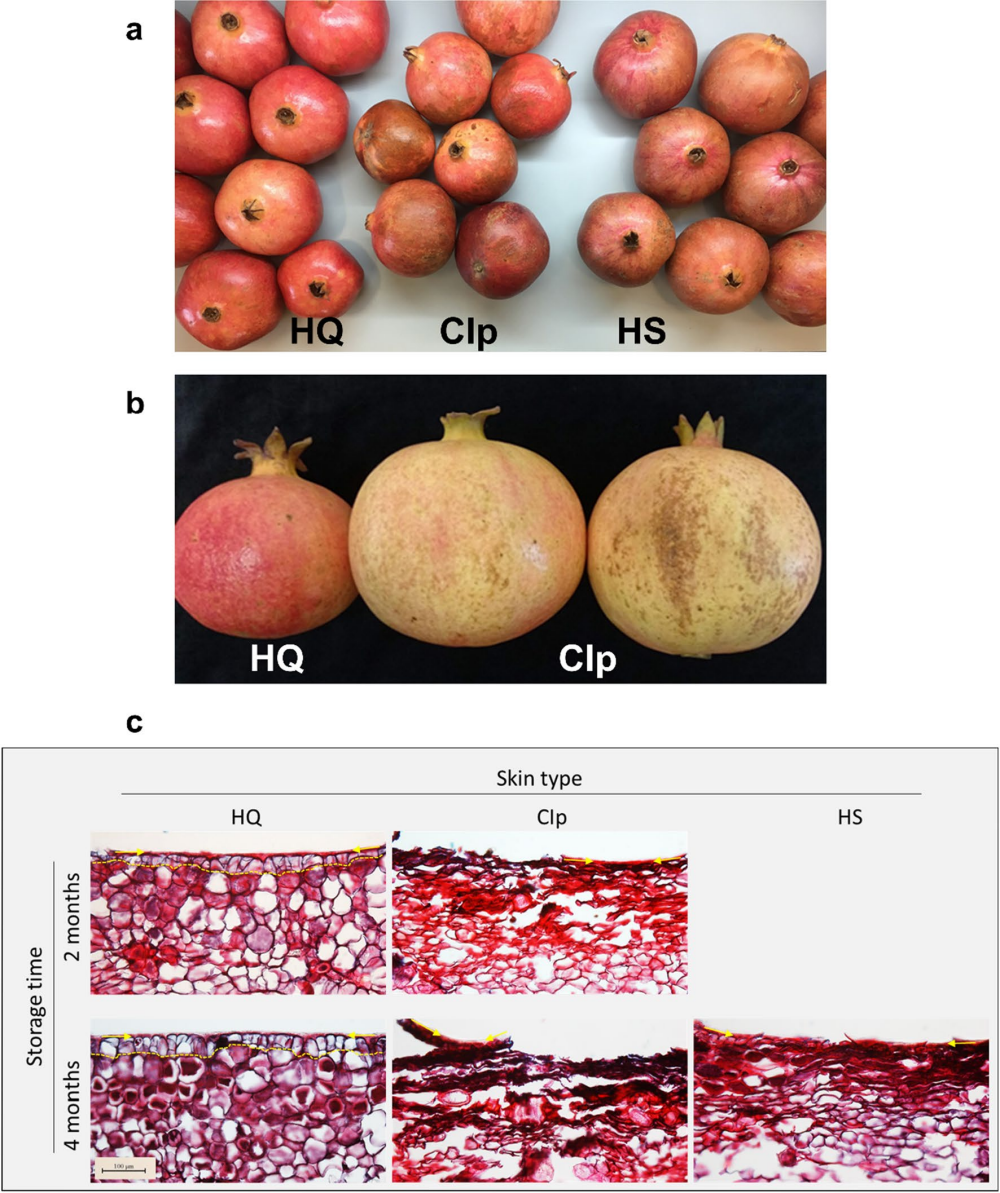
Physiological blemishes on pomegranate skin following 3–4 months of storage at 7 °C in Xtend bags with modified atmosphere. Fruit were collected at maturity (**a**), and at prematuration stage of the skin (**b**). Anatomy of the skin physiological blemishes taken from the mature fruit after 2 or 4 months in storage (**c**). The epidermal cells are underlined with a dashed line. The cuticle layer overlying the epidermal cells is marked with arrows. Each frame demonstrates skin of one representative fruit out of three independent fruit. Bar = 100 µm. *HQ* smooth, shiny and fully colored skin of high quality, *CIp* chilling injuries of the skin, appearing as dark brown regions, *HS* husk scald, appearing as loss of red pigment intensity and development of brownish tint, together with peel hardening.

Experiment 2 was aimed to monitor the changes occur in fruit peel during storage time, prior to CI development. Fruit were picked manually at two time points: early harvest, on 8 Oct 2018, prior to full fruit maturity [premature (PM) fruit] when the fruit color has a greenish-reddish tint, and at maturity, on 25 Oct 2018 [mature (M) fruit] when the fruit fully exhibits the characteristic red color (demonstrated in Fig. 1a, HQ fruit).

PM and M fruit of similar size were collected from inner branches, avoiding the southern side of the tree that is exposed to high daily radiation. The fruits were brought directly from the orchard to the Volcani Center. Fruit of each type were placed in Xtend bags (code: 815-PG28/m for 5 kg), and were stored at 7 °C. For each fruit type, a total of 60 fruit were divided in 15 Xtend bags, four fruit per a bag. One bag was considered one replicate. Fruit were sampled at specific time points: day of harvest, time 0 (t0), after 1 week of storage (w1), and after 1, 2 and 3 months of storage (m1, m2, m3, respectively). At each time point, three bags, each with four fruits, were sampled for each fruit type.

The peel of pomegranate fruit consists of an inner thick spongy white tissue, and an outer thin red skin made up of epidermis cells covered by cuticle. All of the analyses, except for the anatomical study, were performed with the colored skin tissue that was carefully removed from the spongy tissue, from all sides of the fruit, using a peeler. Skin samples were collected for each biological replicate were pooled from all of the four fruits in each bag.

### Skin anatomical study

Samples of the pomegranate surface (blocks of 4 × 3 × 3 mm) were fixed in FAA (50% ethanol, 5% acetic acid and 3.7% formaldehyde, v/v, in water), dehydrated in an ethanol/xylene series and embedded in paraplast (Surgipath Paraplast Plus, Leica Biosystems Richmond Inc., USA) according to standard methods^26^. Tissue Sects. (20 µm) were stained with Safranin-O/Fast green (Sigma Chemicals, Israel) for examination of tissue morphology^27^. Sections were observed under a light microscope (Leica DMLB, Germany) and images were displayed on a monitor through a CCD camera (Leica DC2000) using the Leica IM1000 program. For each treatment, three independent fruit were examined, each was sectioned at three locations along the affected area.

### Determination of AT and phenol content in the skin

Total AT content was determined based on Markham and Ofman (1993)^28^. Skin samples, 200–300 mg, were ground in liquid nitrogen, followed by extraction in 1 mL of water:methanol:acetic acid (5:11:1, v/v/v) solution for 1 h at 4 °C. The mixture was vortexed and separated by centrifugation for 10 min at 14,500 *g* and 4 °C, and the pellet was re-extracted in the same manner. The supernatants containing AT were combined and read in a spectrophotometer (UV-2401PC Shimadzu, Japan) at 520 nm. Analysis was performed with three biological replicates, each with three technical replicates.

TPC was determined in skin extract consisting of 50 mg vacuum-dried powdered samples homogenized in 500 µL ddH_2_O. The homogenates were centrifuged at 5000 *g* for 15 min to separate the liquid extract from the solids, and the supernatants were held at − 20 °C until use. The peel extract was diluted 1:100 and 10 µL was added to the well of a 96-well plate with 50 µL of 10% Folin–Ciocalteu reagent (#F9252, Sigma Chemicals) (w/v in water). After 3 min incubation at room temperature, 40 µL of 7.5% Na_2_CO_3_ (w/v in water) was added to each well, mixed gently and incubated for 40 min at 37 °C. Analysis was performed with three biological replicates, each with three technical replicates. Reactions were read at 760 nm using the Synergy H1 Hybrid Multi-Mode Microplate Reader (Biotek, Fisher Scientific, USA). TPC values were calculated using a standard curve of 0–0.5 g L^−1^ gallic acid (#G7384, Sigma Chemicals), and were expressed as g kg^−1^ gallic acid equivalents (GAE) on a dry weight basis.

Profiling of individual HT and AT metabolites was done in the laboratory of Rachel Amir, Migal, Israel^12^. Identification and quantification of punicalagin isomers, gallic acid and ellagic acid were achieved by comparing the retention time and standard curves of the authentic standards (Sigma-Aldrich, Rehovot, Israel). Unidentified HTs were quantified based on the corresponding chromatogram peak area. Level of the mono- and diglucoside (G and dG, respectively) forms of the major pomegranate ATs was quantified as follow: authentic standards were used for delphinidin-3,5-dG (D-3,5-dG), and cyaniding-3,5-dG (C-3,5-dG) (Sigma Aldrich, St. Louis, MO). D-3-G, C-3-G and pelargonidin glucosides (P-3-G and P-3,5-dG) were quantified based on the corresponding chromatogram peak area.

### Antioxidant capacity of the skin

Two methods were used to analyze the antioxidant capacity of pomegranate skin: ferric reducing ability of plasma (FRAP) and 2,2-diphenyl-1-picrylhydrazyl (DPPH) radical-scavenging activity^15,29^. The FRAP method was performed according to^30^. It measures the ferric-reducing ability of plasma at low pH. An intense blue color is formed when the ferric–tripyridyltriazine (Fe^3+^–TPTZ) complex is reduced to the ferrous (Fe^2+^) form, which is read at 593 nm. The reaction was performed in a 96-well plate and included 15 µL skin extract (prepared as described for TPC determination) diluted 1:150, and 285 µL reagent solution [25 mL of 300 mM pH 3.6 acetate buffer, 2.5 mL of 10 mM Fe^3+^–TPTZ (#T1253, Sigma Chemicals) and 2.5 mL of 20 mM FeCl_3_ (#157740, Sigma Chemicals)], and was read following 4 min incubation at 25 °C in a microplate reader as above, at 595 nm.

DPPH radical-scavenging activity was determined according to Wissam et al. (2012)^31^ in a 96-well plate. The reaction consisted of 15 µL skin extract (prepared as above) diluted 1:100 with water, and 285 µL of 100 µM DPPH (#D9132, Sigma Chemicals) in methanol. After incubation with shaking at 25 °C for 15 min, recordings were performed at 517 nm at 3-min intervals in the microplate reader.

For both FRAP and DPPH methods, the blank reaction was performed with 15 µL methanol. Analyses were run on three biological replicates and three technical replicates. The skin antioxidant capacity was calculated using a standard curve of 0.1–0.7 mM 6-hydroxy-2,5,7,8-tetramethylchroman-2-carboxylic acid (Trolox, #238813, Sigma Chemicals) in methanol. Units were expressed as mmol kg^-1^ Trolox equivalents (TE) on a dry weight basis.

### RNA extraction and quantitative reverse transcription PCR (RT-qPCR)

Total RNA was extracted according to Ginzberg et al. (2009)^32^; cDNA was synthesized using the QuantiTect Reverse Transcription Kit (QIAGEN GmbH, Germany), and ABsolute™ Blue QPCR SYBR Green ROX Mix (Thermo Scientific, USA) was used for qPCR according to the manufacturer’s protocol with specific primers (Supplementary Table S1). Gene expression data was quantified using a standard curve—A mixture of the tested cDNA samples at equal amounts was diluted 1:30, 1:240, and 1:1920 and used in each qPCR with the specific primers. Each RT-qPCR was performed with three biological replicates, each with three technical replicates. Values in each sample were normalized to the expression levels of *PHOSPHOGLYCERATE KINASE* (*PGK*) as the reference gene^23^.

### Statistical analysis

Data were analyzed for statistical significance by Student’s *t*-test using JMP software (http://www.jmp.com). Values presented are averages of three biological replicates ± SE. Different letters indicate significant differences among means by Student’s *t*-test (*P* < 0.05). When multiple comparisons were made on the same chart, different fonts—uppercase, lowercase or Greek letters—were used for each comparison.

## Results

### Chilling injury of the peel (CIp)

The skin of pomegranate fruit that were collected from the packing house following 4 months’ storage in Xtend bags at 7 °C exhibited imperfections. These were superficial physiological defects of two types—dark brown regions covering up to 40% of the fruit surface and were defined as chilling injuries of the peel, CIp (Fig. 1a), and regions that appeared as loss of red pigment intensity together with the development of a brownish tint and peel hardening, and were defined as HS (Fig. 1a). Both CIp and HS were limited to a few upper layers of the peel, and were not evident in the spongy tissue, the inner membranes or the aril compartments (Supplementary Figure S1). Fruit with skin of high quality (HQ fruit) appeared smooth and shiny, with an intense color (Fig. 1a). Overall, 52% of the fruit were of the HQ-skin type, and 27% and 21% were of CIp- and HS-skin types, respectively. Note, CIp and HS were mutually exclusive, and were not displayed on the same fruit. These values demonstrate the severity of the phenomenon of deterioration in pomegranate appearance under prolonged storage (4 months), especially in light of the fact that all fruit were sorted for high export quality prior to storage.

Anatomical analysis of the fruit surface was performed to better describe the phenomenon. Peel samples were taken from 4-month-stored fruit, from regions exhibiting HQ, CIp and HS skin types (Fig. 1c). These were compared to peel samples from 2-month-stored fruit that exhibited an early stage of CIp development; at this time, less than 10% of the fruit were affected and the CIp appeared as patches of low severity and limited size. The HQ skin demonstrated one layer of compact organized epidermis cells with an overlying cuticle, and an intact and compact subepidermal tissue, after both 2 months and 4 months of storage. CIp disorder exhibited a broken cuticle layer with underlying degenerated cells; the damage was limited to the outermost layers after 2 months’ storage (Fig. 1c, skin type CIp, upper panel), whereas after 4 months’ storage, its severity increased and the underlying tissues were affected as well (Fig. 1c, skin type CIp, lower panel). HS skin disorder developed only after prolonged storage, and showed breaks in the epidermis and cuticle, with underlying degenerated cells; however it was limited to the very outer layers of the skin, and the inner layers seemed unaffected (Fig. 1c, skin type HS, lower right panel), in contrast to the CIp disorder.

### Antioxidant activity in HQ-, CIp-, and HS-skin types

**Figure 2:**
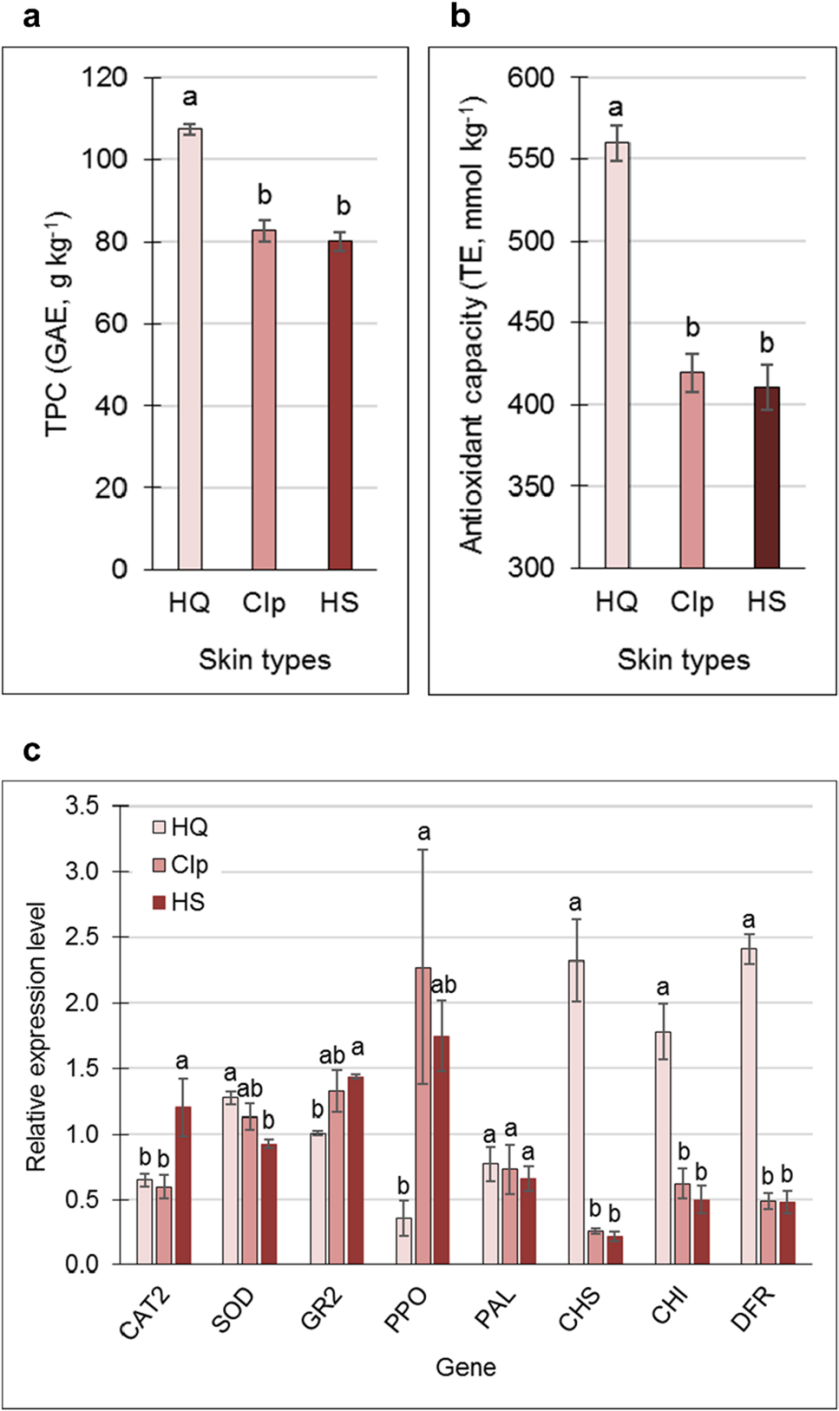
Total polyphenol content (TPC) (**a**), antioxidant capacity (**b**), and the expression of antioxidant-related genes and key anthocyanin-biosynthesis genes (**c**) of skin types shown in Fig. 1. TPC was determined using the Folin–Ciocalteu reagent, and is given as gallic acid equivalents (GAE). Antioxidant capacity was determined using the FRAP method, and is given as Trolox equivalents (TE). Values are averages of three biological replicates ± SE. Data were analyzed for statistical significance among means by Student’s *t*-test; different letters indicate significantly different values (*P* < 0.05).

As phenolics are known to contribute to the antioxidant capacity of pomegranate peel and juice^7,29^, and may enhance skin tolerance to storage conditions, TPC was monitored in HQ-, CIp-, and HS-skin types. Results indicated a significant reduction in TPC in the damaged skin types compared to the HQ skin (Fig. 2a). Accordingly, antioxidant capacity was reduced in CIp and HS skin compared to the HQ skin (Fig. 2b, and Supplementary Figure S2).

To evaluate additional factors that may be involved with skin antioxidant capacity, the expression of genes known to counteract oxidative stress was monitored. Relative transcript levels of *GLUTATHIONE REDUCTASE 2* (*GR2*) and *POLYPHENOL OXIDASE* (*PPO*) were upregulated in CIp and HS skin compared to the HQ skin, albeit not always significantly (Fig. 2c). *CATALASE 2* (*CAT2*) transcript level was upregulated only in the HS skin, whereas the reverse expression profile was found for *SUPEROXIDE DISMUTASE* (*SOD*): low in HS skin and high in HQ skin (Fig. 2c). Expression of key genes in phenylpropanoids biosynthesis pathway—*CHS*, *CHI* and in ATs pathway *DFR*—which could also contribute to the antioxidant capacity of the skin, was strongly downregulated in CIp and HS skin compared to the HQ skin, whereas the expression level of *PHENYLALANINE AMMONIA-LYASE* (*PAL*) was similar for all skin types (Fig. 2c).

### Antioxidant capacity of the skin of early- and late-harvested stored fruit

The overall data described above demonstrated the differences and similarities between storage-induced CIp and HS skin imperfections and HQ skin. However, these were monitored when the damage was already noticeable. To understand the events that occur in the skin prior to its visible breakdown, a controlled experiment was conducted to characterize skin antioxidant capacity during storage. Pomegranate fruit were collected at two time points: early harvest, prior to fruit maturity (PM fruit), when fruit color had a greenish-reddish tint, and at maturity (M fruit) when fruit fully exhibited the characteristic red color. The PM fruit were expected to exhibit higher sensitivity to the storage conditions than the M fruit^11^. Fruit skin was sampled at time of harvest (t0), and the remaining fruit were then stored in Xtend bags at 7 °C, and their skin was sampled after 1 week in storage (w1), and after 1, 2 and 3 months in storage (m1, m2, m3, respectively). At the m3 sampling, the PM fruit skin exhibited CIp that appeared as brown regions on a yellow-tinted background (Fig. 1b).

**Figure 3:**
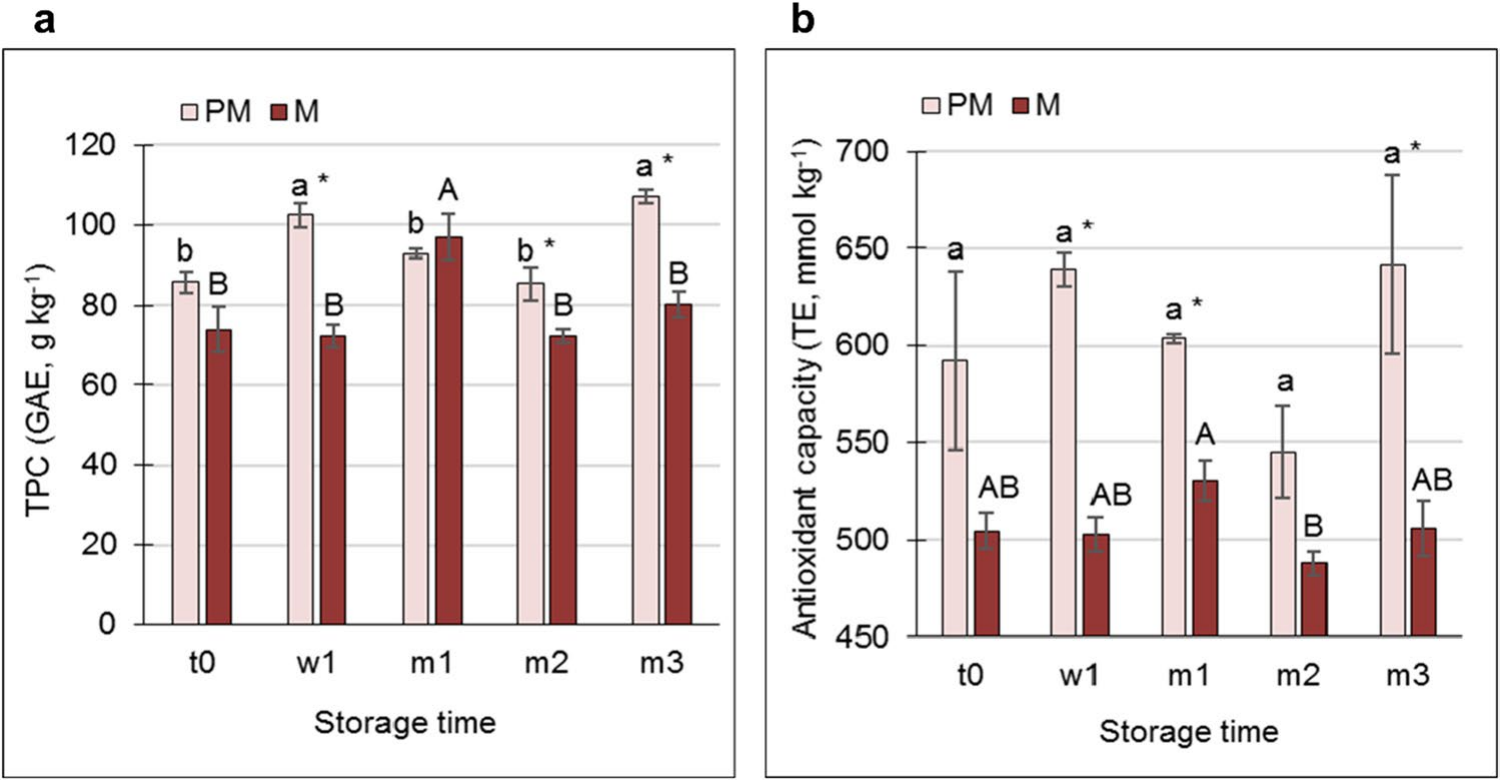
Total polyphenol content (TPC) (**a**) and antioxidant capacity (**b**) in the skin of premature (PM) and mature (M) fruit at harvest (t0), after 1 week in storage (w1), and after 1, 2 and 3 months in storage (m1, m2, m3, respectively). TPC was determined using Folin–Ciocalteu reagent, and is given as gallic acid equivalents (GAE). Antioxidant capacity was determined using the FRAP method, and is given as Trolox equivalents (TE). Values are averages of three biological replicates ± SE. Statistical analysis was performed by Student’s *t*-test (*P* < 0.05). Significant difference among means between time points is indicated by different lowercase letters for PM samples, and uppercase letters for M samples. Asterisks indicate significant differences between PM and M values for the same time point.

A transient increase in TPC in the PM skin was recorded at w1, and then at m3, whereas for the M fruit, this occurred at m1. However, interestingly, TPC values were significantly higher in the PM skin vs. M skin at the longest storage points, m2 and m3 (Fig. 3a). Similarly, the antioxidant capacity in skin from PM fruit was mostly significantly higher than that in the M skin (Fig. 3b, and Supplementary Figure S3).

### Profiling HT in the skin of early- and late-harvested stored fruit

**Figure 4:**
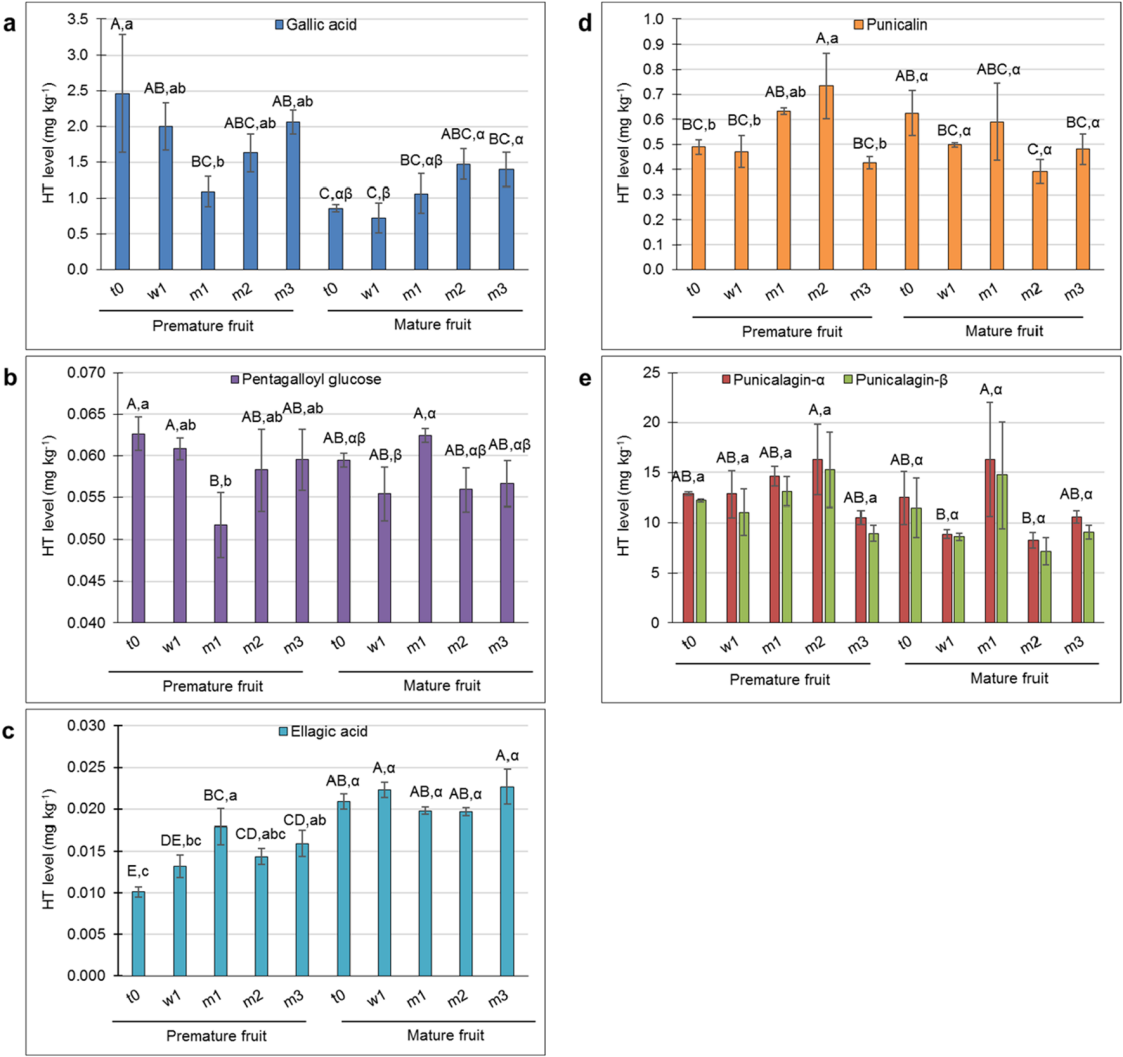
Contents of six members of the hydrolyzable tannins (HT) in the skin of premature and mature fruit at harvest (t0), after 1 week in storage (w1), and after 1, 2 and 3 months in storage (m1, m2, m3, respectively). (**a**) Gallic acid, (**b**) Pentagalloyl glucose, (**c**) Ellagic acid, (**d**) Punicalin, (**e**) Alpha- and beta-Punicalagin. Values are averages of three biological replicates ± SE. Different letters indicate significant differences among means by Student’s *t*-test (*P* < 0.05): uppercase letters for both peel types and all time points, lowercase letters for premature fruit at different time points, and Greek letters for mature fruit at different time points.

Punicalagin isomers are the phenolic metabolites involved in peel antioxidant activity^15,29^. Accordingly, HT profiling was performed (Supplementary Figure S4) to determine the association of punicalagin and its precursors to the differential response of PM- and M-fruit skin to storage conditions. The level of gallic acid, the first metabolite in the HT-specific pathway, was significantly higher in PM skin than in M skin at t0 (Fig. 4a). Its level decreased slightly (but not significantly) in the PM skin during storage (t0 compared to m3), whereas its level increased slightly (but not significantly) in M skin during storage. Nevertheless, overall, the level of gallic acid was maintained at a higher level in the skin of PM fruit during storage compared to M skin, albeit not significantly (Fig. 4a). The reverse trend was observed for ellagic acid level: significantly lower in PM fruit compared to M fruit at t0, and although its level significantly increased in the former during storage, overall, ellagic acid level was significantly higher in M fruit during storage (Fig. 4c). No differences between skin samples or time points were detected for pentagalloyl glucose (Fig. 4b), an intermediate in the pathway between gallic acid and ellagic acid. Note that the levels, in terms of mg kg^-1^, were very low for ellagic acid and pentagalloyl glucose compared to the other HT metabolites.

The level of punicalin (Fig. 4d) increased significantly in PM skin up to m2 and then dropped to its initial values as storage progressed. In M skin, punicalin levels remained similar throughout storage. Punicalagin was the main HT in the pomegranate skin (Fig. 4e); its level was the same in PM and M skin, with no change during the storage. This was true for both punicalagin isomers.

In addition to the known HT metabolites (Fig. 4), some unidentified HTs were detected (Supplementary Figures. S4 and S5). Most of them exhibited higher levels in PM skin than in M skin, both at harvest and during storage.

Summing the HT values, it could be concluded that PM skin contains slightly higher levels of HT than M skin, and that the HT values for each skin type were similarly maintained during storage.

### Total AT and profiling of AT metabolites in the skin of early- and late-harvested stored fruit

Skin AT accumulate largely toward harvest. This was nicely demonstrated by the skin of PM fruit, which was harvested 2 weeks before the optimal time, and contained a significantly lower level of total AT than the skin of M fruit (Supplementary Figure S6, t0). During storage, AT level in the skin of M fruit increased significantly (66%, compare t0 to t3), whereas in PM skin, there was only a slight, nonsignificant increase.

**Figure 5:**
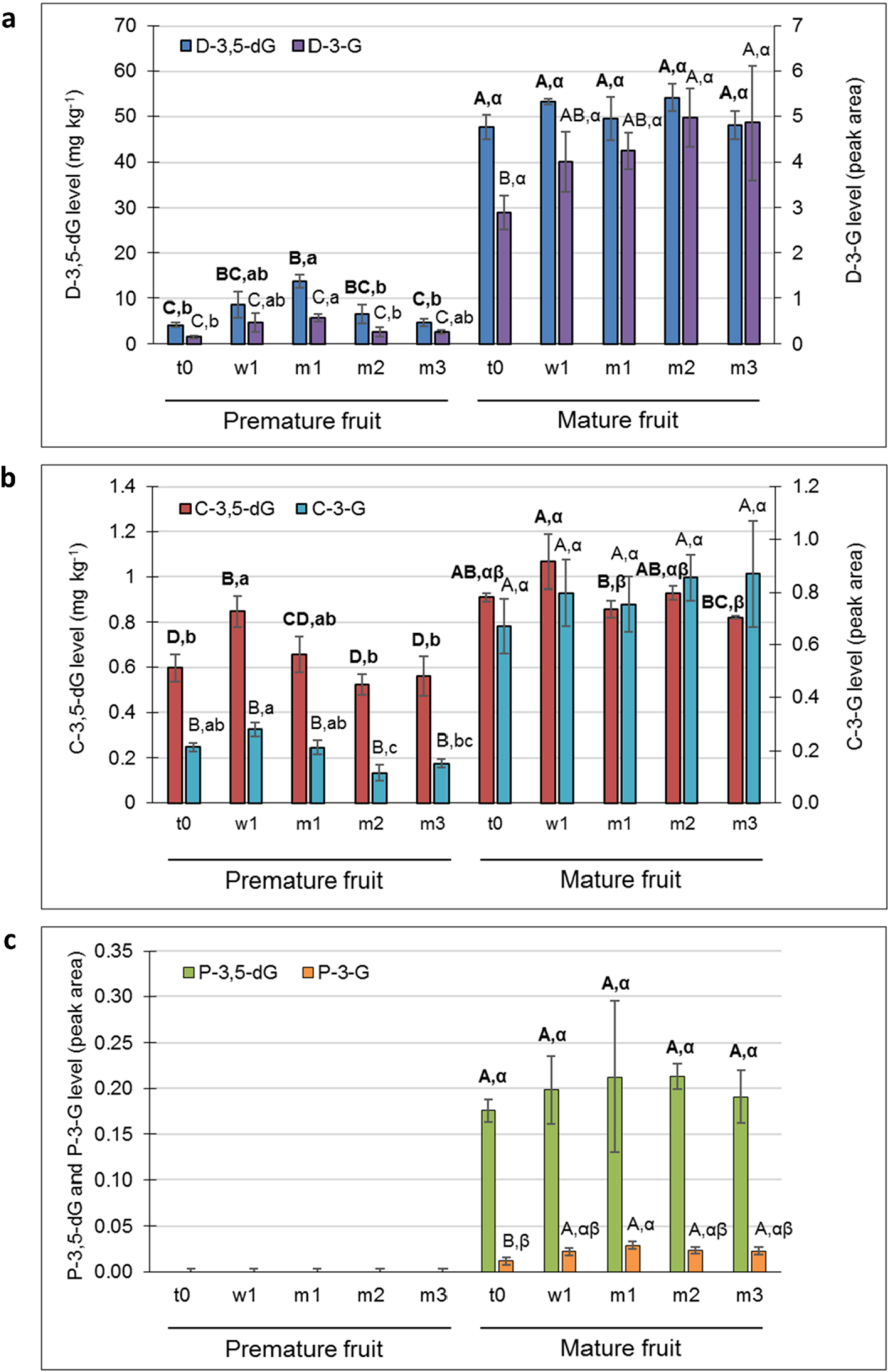
The mono- and diglucoside (dG) forms of the major anthocyanins: (**a**) delphinidin (D-3-G and D-3,5-dG), (**b**) cyanidin (C-3-G and C-3,5-dG), and (**c**) pelargonidin (P-3-G and P-3,5-dG), in the skin of premature and mature fruit. See Fig. 4 for abbreviations and letters of significance; bolded letters refer to the dG form.

Levels of the mono- and diglucoside (G and dG, respectively) forms of the major AT delphinidin (D-3-G and D-3,5-dG), cyanidin (C-3-G and C-3,5-dG), and pelargonidin (P-3-G and P-3,5-dG) in the fruit skin followed a similar pattern (Fig. 5). The levels of AT glucosides were significantly lower in the skin of PM fruit compared that of M fruit, both at harvest and during storage; pelargonidin was totally absent in PM skin. In the PM skin, there was a slight but not significant increase in delphinidin and cyanidin levels at the beginning of the storage period (w1 and m1), but then their levels dropped (Fig. 5a,b). No significant change was detected in the skin of M fruit during storage.

### Gene expression in the skin of early- and late-harvested stored fruit

**Figure 6:**
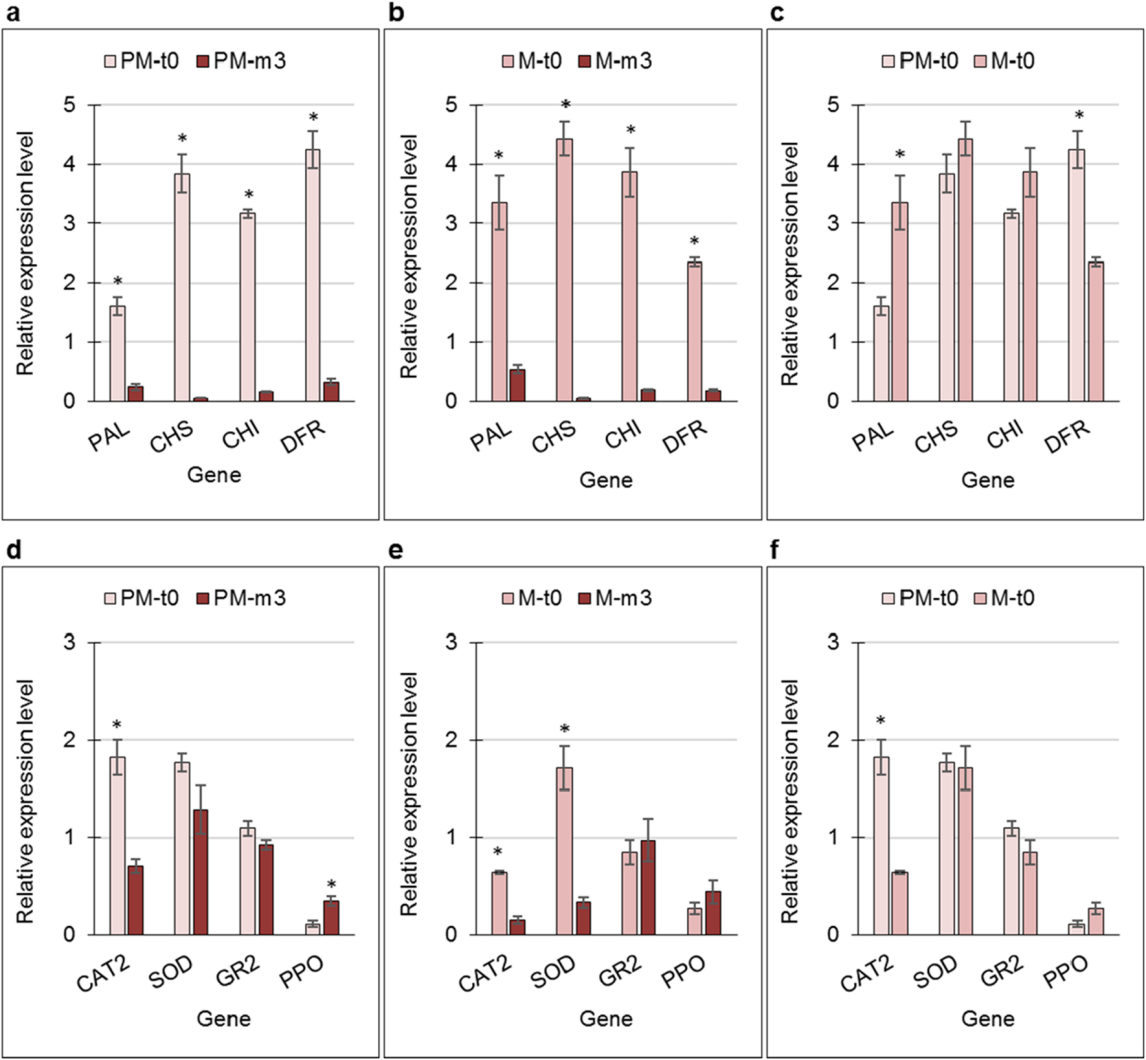
Expression of key anthocyanin-biosynthesis genes (**a**–**c**) and antioxidant-related genes (**d**–**f**) in the skin of premature (PM) and mature (M) fruit at harvest (t0), and after 3 months in storage (m3). Comparisons were made between t0 and m3 for PM (**a**,**d**) and for M fruit (**b**,**e**), and between t0 of PM and M fruit (**c**,**f**). Values are averages of three biological replicates ± SE. Asterisks indicate significant difference among means for each gene by Student’s *t*-test (*P* < 0.05).

To complement the metabolite data, a gene-expression study was conducted, monitoring key genes in AT biosynthesis and those coding for antioxidant activity. Gene expression was compared between samples at harvest (t0) and after prolonged storage (m3) for PM- and M-fruit skin separately, and between PM and M fruit at harvest. There was a dramatic downregulation of AT-related genes—*PAL*, *CHS*, *CHI*, and *DFR*—during storage, in both skin types (Fig. 6a,b). Comparing the t0 data of the two skin types indicated reduced *PAL* and increased *DFR* expression in PM skin compared to M skin (Fig. 6c).

The expression of antioxidant-related genes that are commonly associated with tolerance to low storage temperatures was monitored in the same skin samples. At harvest (t0), *CAT2* expression level was significantly higher than after prolonged storage (m3), in both PM- and M-fruit skin (Fig. 6d,e), and its expression was significantly higher in the PM skin vs. M skin at t0 (Fig. 6f). The expression level of *SOD* was similar in both skin types at t0 (Fig. 6f), but was strongly downregulated following prolonged storage in the skin of M fruit (Fig. 6e), whereas no change was observed in the skin of PM fruit (Fig. 6d). The expression level of *GR2* was similar for both skin types at all time points tested. The expression level of *PPO* was upregulated in the skin of PM fruit following prolonged storage (m3) (Fig. 6d), and only slightly, and nonsignificantly increased in the skin of M fruit.

## Discussion

Prolonged cold storage (> 3 months, 7 °C) promoted the occurrence of brown patches on the skin of pomegranate cv. ‘Wonderful’, although the fruit was held in MA Xtend bags (Fig. 1a). This CIp was manifested as physiological necrosis of the epidermis and hypodermal cortical tissues (Fig. 1c). It was suggested that browning disorder may be due to the enzymatic oxidation of o-dihydroxyphenols to quinone compounds^9^; however the primary cause for the oxidation remained unclear.

The HQ-skin type was resistant during prolonged storage, with no visible anatomical changes after 4 months (Fig. 1c). This raises the questions: Can we identify fruit with HQ-skin type prior to storage? If so, it would enable development of a fruit-sorting system and storage protocol in which fruit with HQ skin could be stored for prolonged periods, if required, while fruit suspected of being sensitive would be marketed immediately. Highly pigmented mature skin is known to be tolerant to storage conditions^7,11^. This is partially true, as PM fruit with weakly pigmented skin was susceptible to the development of CIp (Fig. 1b); however fully mature red skin was not totally immune (Fig. 1a), and 48% of the fruit were affected by CIp or HS. Hence, pigment intensity is not the sole criterion for storage-resistant HQ skin.

The effect of harvest time on the fruit’s chilling response is evident in the internal tissues (the spongy tissue of the peel and the inner membranes that line the aril compartments)^33^. Early-harvested fruit have been found to be extremely chilling sensitive, whereas late-harvested are relatively tolerant^33^. Hence some maturity-related factor determines fruit longevity in storage. To elucidate the underlying molecular mechanism, an RNA-Seq analysis was performed, comparing inner membrane tissues from early- and late-harvested fruit on harvest day and after 2-week exposure to a cold quarantine treatment at 1 °C^11^. The specific functions affected by chilling were: 'starch degradation', 'raffinose', 'ethylene', 'jasmonate', 'abiotic heat stress' and 'RNA regulation of transcription', including AP2/EREBP, bHLH, homeobox, HSFs, MYBs and WARKYs, all transcription factors known to be involved in controlling plant stress responses. In particular, functions related to chilling tolerance of fully mature (late-harvested) fruit included upregulation of jasmonate- and ethylene-signaling members, α- and β-amylase gene members, and several galactinol synthase and stachyose synthase transcripts, which may all be involved in the increase in soluble solutes content that contributes to cold tolerance; finally, small heat-shock proteins related to stress were upregulated as well. These were reviewed in^34^. However these important data refer to the inner membranes of the fruit, and it is not clear how they can be used to acquire pomegranate with increased resistance to CIp.

### Do high TPC and PPO upregulation promote the development of CIp?

Below critical temperatures, membrane microviscosity increases, with a consequent loss of its semi‐permeability and cellular compartmentalization^35,36^. To counteract this, free phenolic compounds scavenge the free radicals released by the oxidation of lipids in cell membranes during the cold stress^37^. On the other hand, the same phenol compounds can be oxidized by PPO, resulting in brown coloration^38^. This is most probably caused by the interaction between PPO, which accumulates in the chloroplast, and phenols stored in the vacuole, following the loss of membrane integrity^35^. Overall, these and similar reports suggest that under cold conditions, a combination of high TPC and high PPO expression increase CIp incidence. This was evident in the skin of 4-month-stored pomegranate. HQ skin was associated with significantly higher TPC compared to CIp- and HS-skin types (Fig. 2a), however *PPO* expression in the former was significantly lower than in the other two (Fig. 2c), and the skin was devoid of CI. This pattern was reversed in the two latter skin types: *PPO* expression was significantly higher in CIp and HS skin than in HQ skin, and therefore associated with the development of chilling-related peel browning (Figs. 1a and 2c). Similarly, increased *PPO* expression in the skin of early-harvested fruit that was stored for 3 months (Fig. 6d, compare m3 to t0) was associated with CIp development on PM skin (Fig. 1b**)**, whereas in M skin that is devoid of CIp, *PPO* expression did not increase (Fig. 6e, compare m3 to t0). Furthermore, TPC level was significantly lower in M skin compared to PM skin at m3 (Fig. 3a). These data are supported by a previous publication in which PPO activity and total phenolic and total tannin contents were associated with the development of HS, although this study used whole peel, not just the skin^39^.

In addition, lower peel browning was associated with a high ratio of PAL-to-PPO activity, obtained following immersion of the pomegranate in arginine solution^38^. This high ratio signifies a high level of phenols and AT, and a low level of PPO activity. Using our data, the calculated *PAL*-to-*PPO* ratio at the transcript level was high for the HQ-skin type compared to a low value for CIp- and HS-skin types (2.18, 0.32 and 0.3, respectively); similarly, the *PAL*-to-*PPO* transcript ratio following 3 months in storage was higher for M skin than for the CIp-susceptible PM skin (1.2 and 0.7, respectively).

**Figure 7:**
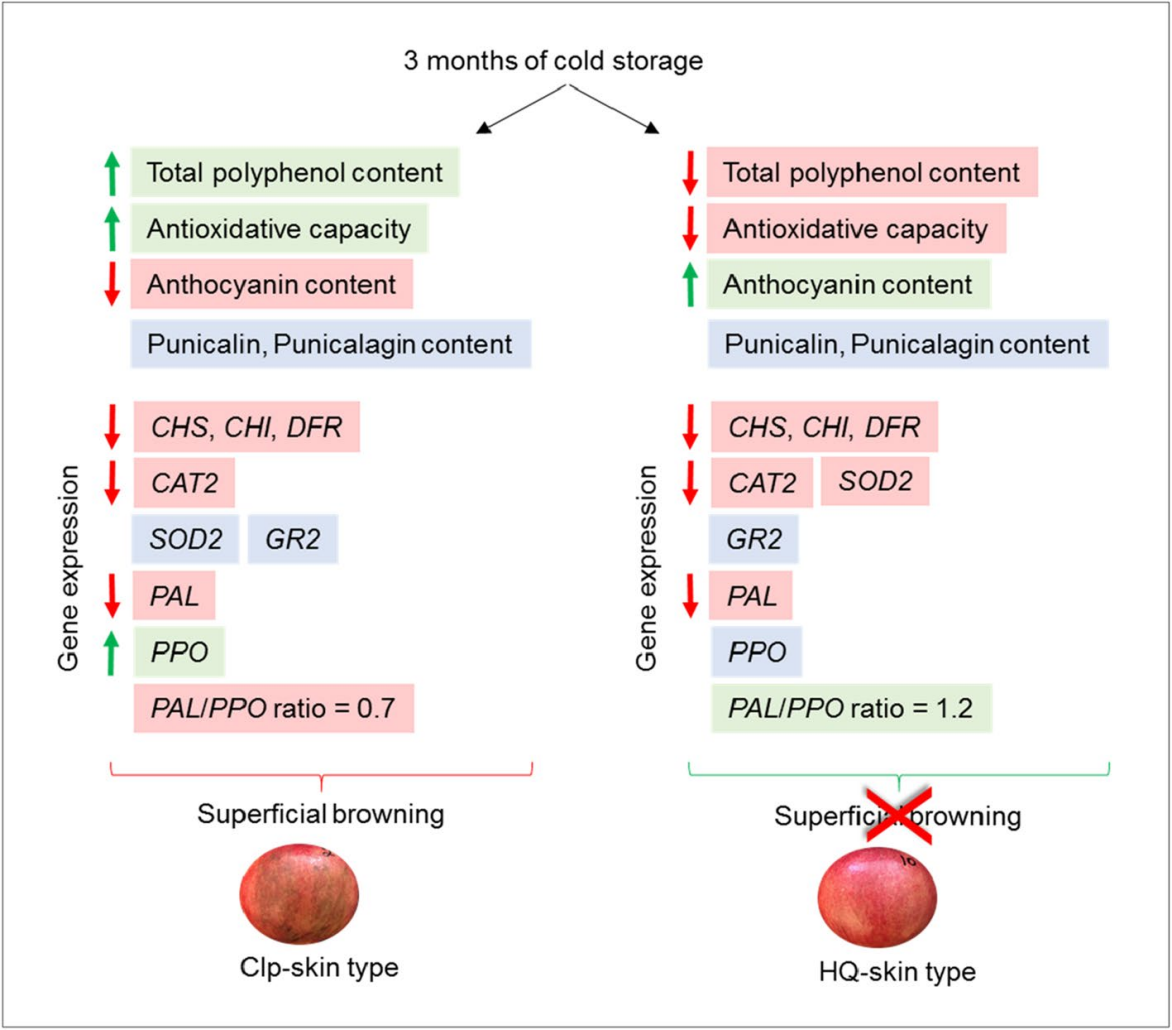
Schematic presentation summarizing the factors involved in promoting chilling injury of the pomegranate peel (CIp). The content of total polyphenols, anthocyanins, punicalin and punicalagin, and antioxidative capacity were compared between premature (PM) skin type that is susceptible for superficial browning and mature (M) skin (Figs. 3, 4, 5 and supplementary Figure S3). Up- and downregulation of genes expression was determined by comparing their level at harvest and following 3 month of storage in PM and M skin types (Fig. 6). Red background represents reduced level; green, induced level; blue, no significant change. Data suggests that high total polyphenol content and low anthocyanins level together with low value for *PAL*/*PPO* ratio promote CIp.

Overall, it is suggested that high TPC combined with high *PPO* expression are associated with CIp development in pomegranate cv. ‘Wonderful’ (Fig. 7). Downregulation of *PPO* expression in the fruit skin during prolonged storage may reduce CIp and HS disorders.

### Antioxidant capacity and related gene expression are not associated with the prevention of CIp development

High TPC has positive and negative aspects. On the one hand, in combination with PPO, high TPC may promote skin browning. On the plus side however, high TPC is associated with high antioxidant capacity of the tissue^15^. This was evident for the skin of HQ fruit, where high TPC was associated with a significant increase in antioxidant capacity, and inversely so for CIp- and HS-skin types (Fig. 2a,b). Similarly, a positive correlation between high TPC and high antioxidant capacity was detected in the skin of stored PM fruit (Fig. 3); however, unexpectedly, the skin of PM fruit was sensitive to CIp development (Fig. 1b). In contrast, the skin of M fruit had lower TPC levels and lower antioxidant capacity than the PM fruit, and was devoid of CIp (Fig. 3). It is suggested that high TPC that is associated with high antioxidant capacity is not sufficient to prevent the development of CIp (Fig. 7). This conclusion is supported by previous analysis of seven accessions indicating that fruit with high antioxidant capacity, high TPC, and high levels of punicalin and AT in their peels are not necessarily resistant to CIp^7^. The only reservation here is that the analyses were performed with whole fruit peel, rather than just the skin.

The expression of genes related to antioxidant activity which are also known to be involved in CI^38^ provided an additional aspect in our examination of the effect of antioxidative potential of the skin on resistance to CIp development. *CAT2* and *GR2* were significantly upregulated in HS skin compared to HQ skin, whereas *SOD* expression showed the reverse pattern (Fig. 2c). However, since these skin samples were already damaged by the cold conditions, it is not clear whether the observed gene expression was in response to the cold stress or to skin degradation. When gene expression was compared between PM and M skin at harvest (t0) and following 3 months of storage (m3), *CAT2* expression level was significantly higher in the PM-skin type, and significantly higher in both skin types at t0 compared to m3 (Fig. 6d–f). *GR2* expression was the same for both skin types and at the two time points; *SOD* expression showed a similar pattern, except for its significantly lower expression in M skin following the prolonged storage (Fig. 6e). Results suggest that storage-induced downregulation of *CAT2* is not associated with CIp development, as its expression level was also reduced in M skin, where no CIp developed. It is probable that *SOD* and *GR2* also are not “players” in CIp development (Fig. 7).

### HT and AT metabolites in PM and M skin and their association with skin tolerance to storage conditions

Data on HT metabolites indicated no significant difference between PM- and M-skin types at t0 or during storage, for either isomer of punicalagin, the major metabolites in pomegranate skin (Fig. 4). The punicalagin remained constantly high in both tissues, and during storage. This in contrast to previous report of a decline in punicalagin level in the peels of several tested accessions^7,8^. The discrepancy could result from a different storage protocol and peel sampling method—our fruit were stored in Xtend bags and we sampled only the skin, not the whole peel.

The level of gallic acid was lower in M skin than in PM skin at t0, and generally lower during storage, albeit not significantly so; the same was found for some unidentified HT (Supplementary Figure S5). As the concentrations of the various HT types were negligible compared to that of the punicalagin isomers, it is suggested that total HT, and in particular punicalagin level, are not associated with CIp occurrence or prevention in the presently tested pomegranate skin. However, although the physiological significance of the unidentified HTs is not yet clear, the outstanding difference between PM and M skins in the levels of the HTs B, C, D and E (Supplementary Figure S5) might be useful as a harvest marker for potential CI sensitivity.

In addition to HT, the AT metabolites are also known to contribute to the antioxidant capacity of a tissue. They also serve as an indicator of peel maturity, and poorly colored fruit/accessions are more sensitive to CI development^7,33^. In the skin of PM fruit, total AT concentration was significantly lower than in M skin (Supplementary Figure S6). This was expected as the PM-skin type was collected from early-harvested fruit when pigment accumulation has not yet peaked. Moreover, total AT concentration increased during storage only in the skin of the late-harvested (M) fruit, whereas in the PM skin, total AT remained unchanged and was at a very low level (Supplementary Figure S6). It is hypothesized that in PM skin, the AT-biosynthesis pathway is not fully activated; once the fruit is detached from the tree, AT biosynthesis “freezes”; in M skin, the pathway is active at harvest, and AT biosynthesis can proceed during storage. In this context, it is notable that the expression of *PAL* was significantly lower in the skin of PM fruit at harvest compared to the skin of M fruit (Fig. 6c).

Interestingly, despite the above discussion, a study of key AT genes’ expression showed strong reduction of *CHS*, *CHI* and *DFR* transcripts in both tissues and during storage. Chilling-induced downregulation of *CHS* and *DFR* was also detected in the inner membrane transcriptome of ‘Wonderful’, and it was suggested that under cold storage (2 weeks at 1 °C), the phenylpropanoid pathway is induced and there is diversion toward the phenol metabolism pathway rather than toward the flavonoid biosynthesis pathway^40^. In the skin, cold regulation of AT and phenylpropanoids may differ, as *PAL* expression is downregulated in this tissue (Fig. 6a,b), whereas it is upregulated in the inner membranes^40^.

Although its biosynthetic pathway is downregulated, AT continued to accumulate in the skin of M fruit during storage (Supplementary Figure S6). It is assumed that in the M skin, enzyme activity was maintained, and AT pigments were stabilized in the vacuole^16^, resulting in the maintenance of a high level of AT during storage. Low AT levels and the inability of PM skin to upregulate the AT-biosynthesis pathway during storage may contribute to the sensitivity of that skin type to CIp development (Fig. 7).

HPLC analysis of the main AT in the skin indicated delphinidin glucosides as the major metabolites, followed by cyanidin and pelargonidin glucosides (Fig. 5). The level of all of these metabolites in the PM skin at harvest and during storage was significantly lower than the corresponding values in M skin. This is in accordance with the data obtained from the determination of total AT.

HT- and AT-specific biosynthetic pathways divert from the shikimate pathway. It has been argued that the two former pathways compete for the same precursor metabolites, and thus exhibit opposite trends of accumulation^12^. A positive correlation between AT and total punicalagin contents in the peel of red pomegranate cv. P.G.116–17 which contains a high level of AT was shown^13^. It was suggested that high activity of the related pathways, including the shikimate pathway, provides sufficient precursors for AT and HT synthesis. However, a negative correlation between ATs and HTs in the outer peel of 33 pomegranate accessions was shown^12^. It suggested a competition for precursors between the HT- and AT-biosynthesis pathways, with 3-dehydroshikimate being the common precursor, partitioning the HT pathway from the shikimate pathway at the stage of shikimate dehydrogenase activity, while the AT pathway is diverted from the downstream pathways of shikimate and the phenylpropanoids^12^.

In the present study, total AT and punicalagin levels were monitored during skin maturation (PM and M skin were compared at t0) and during 3 months in storage. In all samples tested, the levels of the punicalagin isomers—the major HT in the skin—remained the same (Fig. 4e), even when AT level increased significantly, by several fold (Fig. 5 and Supplementary Figure S6). This suggests that here, accumulation of AT did not interfere with maintaining an even level of punicalagin. Yet, it is noted that the levels of gallic acid (Fig. 4a) and of additional unidentified HTs (Supplementary Figure S5) were reduced, although the total change may have been quantitatively insignificant.

## Conclusion

It is well known that pomegranate fruit should be harvested at their mature stage with dark-pigmented skin, because early-harvested, poorly colored fruit are more susceptible to CIp development. However, pigment intensity is not the sole criterion for CIp resistance, nor are high HT concentration, or the high antioxidant capacity associated with high TPC. Results suggest that high TPC combined with high PPO activity are associated with CIp development in pomegranate. Hence, downregulation of *PPO* expression in the skin of pomegranate during prolonged storage may reduce CIp disorder.

## Supplementary Information

Supplementary Information 1.

Supplementary Information 2.
