# Supplementary material for: Metabolic changes in pomegranate fruit skin following cold storage promote chilling injury of the peel
Source: Sci Rep. 2021 Apr 28;11:9141. doi: 10.1038/s41598-021-88457-4 (PMC8080622; doi:10.1038/s41598-021-88457-4)
Supplement: Supplementary file 1 — Supplementary Information 1. [file 41598_2021_88457_MOESM1_ESM.pdf]

Supplementary information

Metabolic changes in pomegranate fruit skin following cold storage promote chilling injury of the peel

Ravi Singh Baghel[1,2], Alexandra Keren-Keiserman[1], Idit Ginzberg[1*]

[1] Institute of Plant Sciences, Agricultural Research Organization, Volcani Center, 68 HaMacabim Road, P.O. Box 15159, Rishon LeZion 7505101, Israel

[2] Present address, Biological Oceanography Division, CSIR-National Institute of Oceanography, Dona Paula-403004, Goa, India

*Corresponding author: e-mail: iditgin@volcani.agri.gov.il

Supplementary Table S1. Primers used in the study.

Gene	Gene ID	Forward primer (5'→3')	Reverse primer (5'→3')	Amplicon size (bp)
PAL	CDL15_Pgr025556	TGAGTCAATCCCTGCAACCT	ATCACGAAGCTCCTGAACCA	192
CHS	CDL15_Pgr026373	AGAAAGAAGTGCGTCGAGGA	ACGCTATGGAGAACGACTGT	113
CHI	CDL15_Pgr025723	AGGAAGAAGCGGCCTTAGAG	CCTCGACAGTAGGTGAAGCT	108
DFR	CDL15_Pgr021399	GAATCAAGGTTCGAAGGGGTC	CGAACATGTCCTCCAAGCTG	109
CAT2	CDL15_Pgr014250	CTTCAAGCCAGCTGGAGATAG	AGACGGGTTGCTAGCTTCTG	177
SOD2	CDL15_Pgr025123	TCACTTGCACGAGTATGGAG	TGGCAATTATGTTTCCCAGGT	134
GR2	CDL15_Pgr023758	TTGACTCCTGTTGCCCTGATG	TTTAACGGCCGGAAGTTTGC	200
PPO	CDL15_Pgr016899	ACGAAAATGCCCAACCTGTC	GGGAACTGCACTCTCGGTAA	199
PGK	CDL15_Pgr007113	ATGCCACCCAGACCATCATT	CCGATAATGGTTGTCACTCC	130
