# Supplementary material for: Metabolic changes in pomegranate fruit skin following cold storage promote chilling injury of the peel
Source: Sci Rep. 2021 Apr 28;11:9141. doi: 10.1038/s41598-021-88457-4 (PMC8080622; doi:10.1038/s41598-021-88457-4)
Supplement: Supplementary file 2 — Supplementary Information 2. [file 41598_2021_88457_MOESM2_ESM.pdf]

Supplementary information

Metabolic changes in pomegranate fruit skin following cold storage promote chilling injury of the peel

Ravi Singh Baghel[1,2], Alexandra Keren-Keiserman[1], Idit Ginzberg[1*]

[1] Institute of Plant Sciences, Agricultural Research Organization, Volcani Center, 68 HaMacabim Road, P.O. Box 15159, Rishon LeZion 7505101, Israel

[2] Present address, Biological Oceanography Division, CSIR-National Institute of Oceanography, Dona Paula-403004, Goa, India

*Corresponding author: e-mail: iditgin@volcani.agri.gov.il

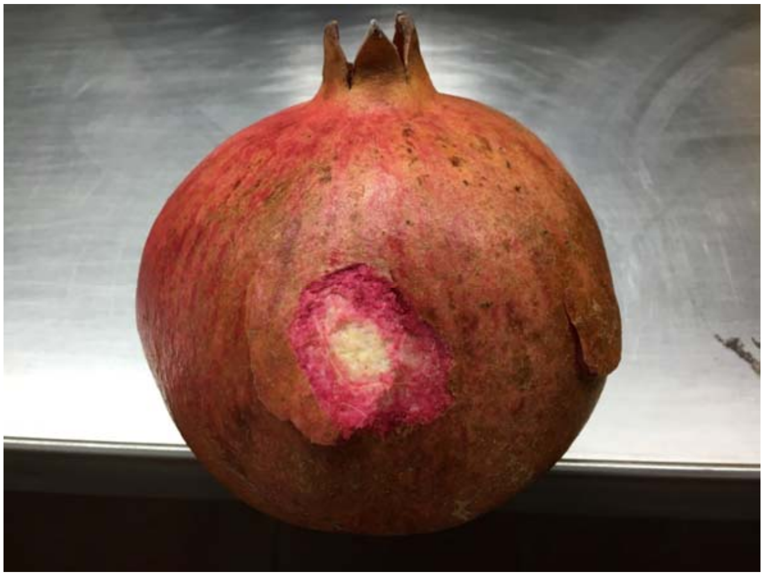

Supplementary Figure S1 Storage-induced chilling injuries of the peel (CIp) are limited to the very outer layers of the peel.

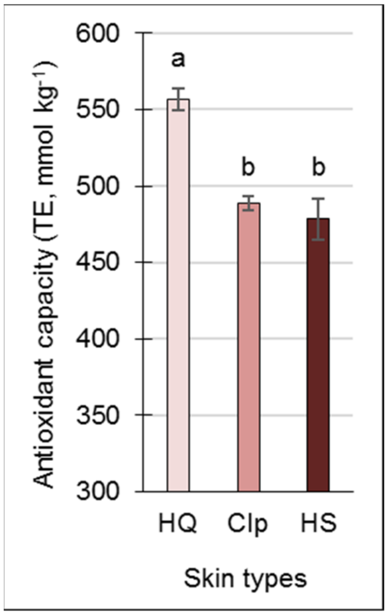

Supplementary Figure S2 Antioxidant capacity in pomegranate skin determined by the DPPH method and given as Trolox equivalents (TE). Skin types are as described in Fig. 1A. Values are averages of three biological replicates ± SE. Data were analyzed for statistical significance among means by Student's *t*-test; different letters indicate significantly different values ($P < 0.05$).

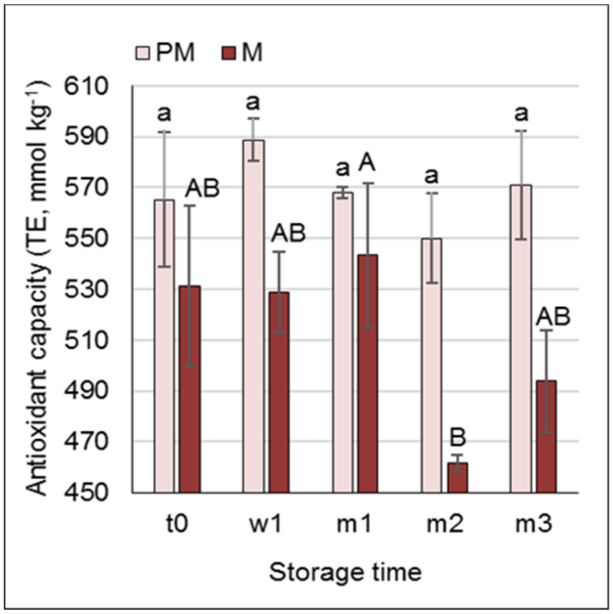

Supplementary Figure S3 Antioxidant capacity in skin of premature (PM) and mature (M) pomegranate fruit at harvest (t0), after 1 week in storage (w1), and after 1, 2 and 3 months in storage (m1, m2, m3, respectively). Antioxidant capacity was determined by the DPPH method and is given as Trolox equivalents (TE). Values are averages of three biological replicates ± SE. Statistical analysis was performed by Student's *t*-test ($P < 0.05$). Significant difference among means between time points is given by different lowercase letters for PM samples, and uppercase letters for M samples.

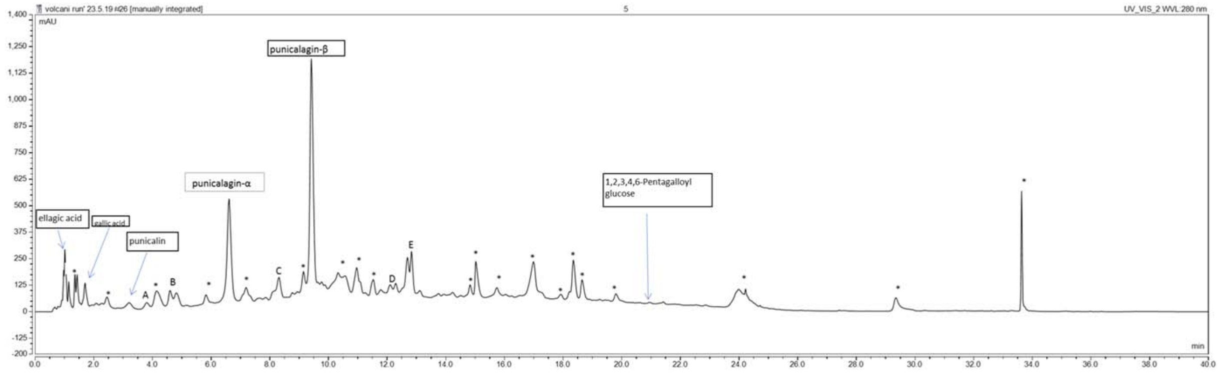

Supplementary Figure S4 HPLC profile of hydrolyzable tannins (HT). Peaks corresponding to identified and unidentified (A–E) HT are labeled.

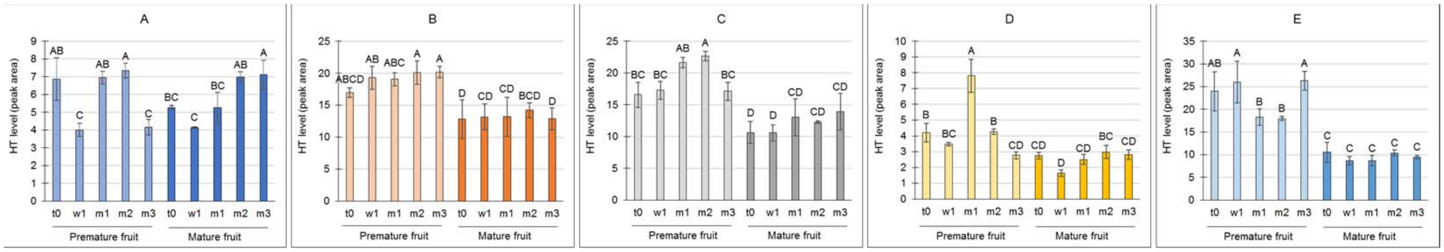

Supplementary Figure S5 Levels of unidentified HT (shown in Supplementary Figure S4) in the skin of premature and mature fruit at the harvest (t0), after 1 week in storage (w1), and after 1, 2 and 3 months in storage (m1, m2, m3, respectively). Values are averages of three biological replicates ± SE. Data were analyzed for statistical significance among means by Student's *t*-test; different letters indicate significantly different values ($P < 0.05$).

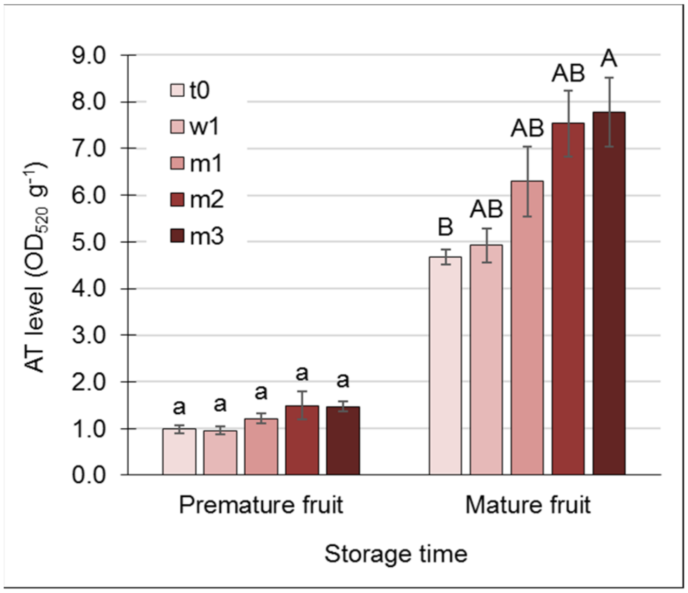

Supplementary Figure S6 Total anthocyanin (AT) in the skin of premature and mature fruit at harvest (t0), after 1 week in storage (w1), and after 1, 2 and 3 months in storage (m1, m2, m3, respectively). Significant difference among means is indicated by different lowercase letters for the premature samples, and uppercase letters for the mature samples.
